# Percolation in Networks of Liquid Diodes

**DOI:** 10.1021/acs.jpclett.3c01885

**Published:** 2023-08-22

**Authors:** Camilla Sammartino, Yair Shokef, Bat-El Pinchasik

**Affiliations:** † School of Mechanical Engineering, Tel Aviv University, Tel Aviv 69978, Israel; ‡ Center for Physics and Chemistry of Living Systems, Tel Aviv University, Tel Aviv 69978, Israel; ¶ Center for Computational Molecular and Materials Science, Tel Aviv University, Tel Aviv 69978, Israel; § International Institute for Sustainability with Knotted Chiral Meta Matter, Hiroshima University, Hiroshima 739-8511, Japan

## Abstract

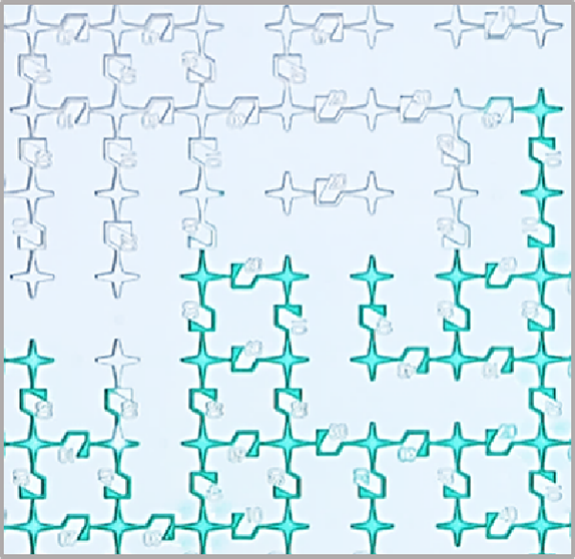

Liquid diodes are surface structures that facilitate the spontaneous flow of liquids in a specific direction. In nature, they are used to increase water collection and uptake, reproduction, and feeding. However, large networks with directional properties are exceptional and are typically limited up to a few centimeters. Here, we simulate, design, and 3D print liquid diode networks consisting of hundreds of unit cells. We provide structural and wettability guidelines for directional transport of liquids through these networks and introduce percolation theory in order to identify the threshold between a connected network, which allows fluid to reach specific points, and a disconnected network. By constructing well-defined networks with uni- and bidirectional pathways, we experimentally demonstrate the applicability of models describing isotropically directed percolation. We accurately predict the network permeability and the liquid final state. These guidelines are highly promising for the development of structures for spontaneous, yet predictable, directional liquid transport.

Percolation theory is used to describe critical phenomena in multiple types of complex physical systems^1^ such as flow through porous or granular media,^2,3^ electrical conductivity,^4^ spreading of fires,^5^ vascular networks,^6^ biomolecular transport,^7,8^ jamming of particulate systems,^9−11^ and even the formation and release of traffic jams.^12^ These phenomena can be described by different percolation models,^1,13^ through the formation of connected clusters and networks. Well-defined, predictable experimental realizations of these models, however, still remain an open field of research, with potential applications in fluidics, electronics, power grids, epidemics, and biology.^14^

To date, experimental efforts have focused on percolation of electrical conductivity,^15−19^ mainly using the bond percolation model. In this model, the network is characterized by the probability *p* of having a bond between two neighboring sites. Similarly, *p*_0_ = 1 – *p* is the probability of having a missing bond, namely, a vacancy. If enough bonds are present, an infinite connected cluster is formed, and the network percolates. The critical probability of existing bonds, *p*_*c*_, defines the threshold between the nonpercolating and percolating phases.^20^

In classic, or random, bond percolation, all bonds are bidirectional. Consequently, percolation through the network is isotropic. In directed percolation, on the other hand, the bonds are directional, and allow transport only in a preferred direction in the network.^21,22^ Thus, such a system is connected anisotropically. An example of such directional transport in nature is seen in the Texas horned lizard. This desert lizard increases its water collection and intake by passive water transport through a network of directional channels between its scales, directing the water to the mouth over distances of a few centimeters.^23,24^ Another, more recently introduced, percolation model corresponds to isotropically directed percolation.^25−29^ There, bonds in opposite directions exist with a total probability *p*_1_, together with bidirectional bonds, with probability *p*_2_, and vacancies with probability *p*_0_ = 1 – *p*_1_ – *p*_2_. This type of percolation is governed by the probability to find a bond between a site and one of its neighboring sites,^29^ which is equal to . Isotropically directed percolation has been used to analyze traffic patterns in New York and London,^30,31^ but more experimental work is needed to further understand the applicability of the theory to real-life situations.

In this study, we introduce an experimental realization of isotropically directed percolation using a network of three-dimensional (3D) printed liquid diodes. The liquid diodes comprise 3D surface structures that promote spontaneous unidirectional liquid flow in the capillary regime.^32^ We design a 2D network made of millimeter-size open channels^32,33^ that set the bonds between neighboring sites. By tuning the geometric features of the liquid diodes and the contact angle (CA) that the flowing liquid creates with the surface, we control the diodicity of the bonds. Namely, whether the flow through a bond is uni- or bidirectional. As a result, we are able to control the number of directional bonds, scan over different values of *p*_*nn*_, switch between different percolation states and find the percolation threshold for each configuration of the network. Establishing a well-defined physical system that enables fine-tuning of percolation parameters enables us to gain new insights into the fundamentals of directional transport phenomena in general,^34^ and isotropically directed percolation specifically. This includes direct measurements of the flow dynamics through the network and studying the influence of the system size and initial conditions of the liquid on its final state and configuration. In addition, controlling the percolation threshold and being able to tune the permeability of a network opens new horizons for designing microfluidic complex networks for mixing and separation,^19,35^ heat transfer^36^ and actuation.^37^

Figure 1 shows the liquid diodes used in this work^33^ and how they are implemented to form 2D disordered isotropically directed networks. Each diode features an asymmetric geometry comprising four main components (Figure 1a,i): the entrance channel (hilla), a central area (bulga), an exit channel (orifice), and, in blue, an ellipsoid-shaped bump (pitch), shown in more detail in Figure 1a,ii. The bulga is aligned perpendicular to the orifice, creating a 90° expansion in the channel’s width. This expansion, together with the pitch, creates a pressure barrier that pins the liquid in the backward direction (left to right). The height of the pitch is defined in terms of the percentage of the channel’s depth, and in our networks, it varies between 10% and 60%, in steps of 10%.

**Figure 1:**
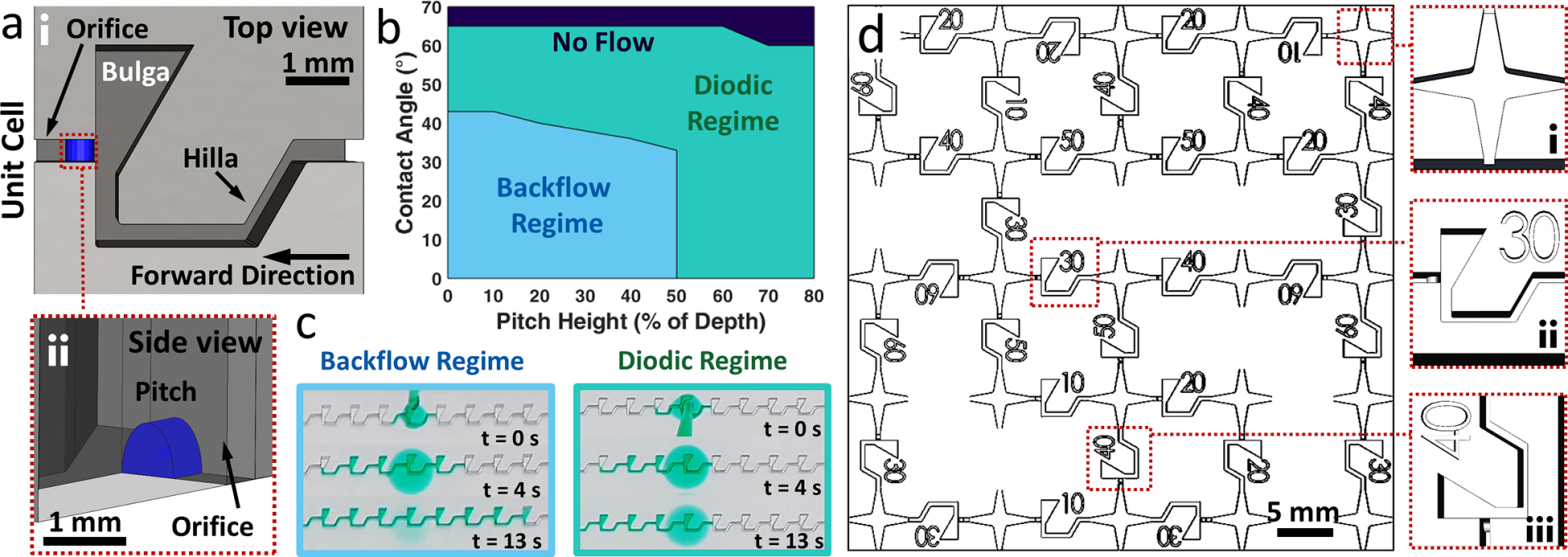
Liquid diodes design and function. (a) Unit cell and main components (i). (ii) Side view of the pitch, an elliptical-shaped bump (blue). The outer wall is made transparent. (b) Flow phase diagram depending on the pitch height and liquid contact angle. Three main behaviors are observed: no flow (dark blue), diodic regime (green), and backflow regime (light blue). (c) Top view of 3D printed channels made of ten consecutive liquid diodes with 0% (left) and 40% (right) pitch height, showing backflow and diodic regime, respectively. (d) Schematics of a representative 5 × 5 isotropically directed network. The insets show the design of (i) a node, (ii) a 30% pitch height bond oriented so that the forward direction is right to left, and (iii) a 40% pitch height bond oriented so that the forward direction is top to bottom.

Different flow regimes arise depending on the combination of the pitch height and the CA of the flowing liquid, summarized in the phase diagram shown in Figure 1b. In green is the diodic regime, where the flow is unidirectional. Figure 1c, right depicts timeframes of liquid flow in a 3D printed channel made of several consecutive unit cells with 40% pitch height and a CA of 45°. By lowering the CA (i.e., increasing the liquid wettability^38^), the diodes start exhibiting flow in the backward direction and the diodicity breaks (Figure 1c, left). The dependence of the diodes’ performance on the pitch height is associated with the local reduction and following expansion of the channel’s depth, caused by the pitch, when the liquid flows in the backward direction.^33^ For example, with a pitch height of 40%, the channel depth is locally reduced to 60% of its original value. After the pitch, the channel depth returns to its full value (100%). This creates an additional pressure barrier when liquid propagates in the backward direction and has to overcome the pitch (see Figure S1). In the topmost part of the phase diagram, in dark blue, we observe no flow in either direction due to the poor wettability of the liquid.

Figure 1d shows an isotropically directed 5 × 5 network comprising diodes of different pitch heights and orientations. We created three networks, 15 × 15 in size, with *p*_0_ values of 0.2, 0.31, and 0.37, featuring, 336, 288, and 264 bonds, respectively, using diodes with pitch heights ranging from 10% to 60%, in steps of 10%. Within a given sample, we have the same number of diodes per pitch height and orientation. Hence, at each site, the probability of a unidirectional bond in each direction is uniform, and the network is isotropic. The distribution of vacancies and diodes, in each direction, is randomized (see Materials and Methods). As we change the CA of the flowing liquid, a fraction of the bonds switches behavior according to the phase diagram in Figure 1b, allowing us to scan over different values of *p*_*nn*_ for each sample of a given *p*_0_. This is illustrated in Figure S2, using schematics of the three samples with different *p*_0_, for increasing *p*_*nn*_ values or decreasing CAs. As *p*_*nn*_ increases, the number of bidirectional bonds, indicated by magenta two-way arrows, increases, creating a larger connected cluster.

We used numerical simulations to predict the flow pattern and the liquid propagation through the network. For each network configuration, we inserted a liquid with a known CA at a specific feeding site and let it propagate spontaneously, until the liquid fronts halt. Once the liquid reached the final state, we extracted the fraction of occupied sites *f*. The simulations were repeated for all of the possible feeding sites along the edges of the network and for all CA values, using a mapping that adjusts the network design according to the phase diagram shown in Figure 1b.

We verified our predictions by flow experiments in the 3D-printed liquid diodes networks. The experiments were conducted in the same fashion as the simulations (see Materials and Methods) and repeated for eight randomly chosen feeding sites, two for each edge of the network, and for each CA.

Figure 2 shows simulations (upper row) and experiments (bottom row) of the liquid final states with increasing *p*_*nn*_ (0.34, 0.51, 0.63) for *p*_0_ = 0.31. The red arrows denote the feeding sites (identical in all cases). When *p*_*nn*_ increases, a larger portion of the network is covered with liquid, a result of the increasing number of bidirectional bonds. Videos S1, S2, and S3 show the propagation dynamics of the three experiments. This leads to a phase transition between a nonpercolating and a percolating state. The liquid distribution is isotropic. Namely, no preferred direction is observed, and the liquid spreads homogeneously in all directions. We find excellent agreement between the simulations and experiments. Yet, a few local failures in the diodes were observed due to pressure buildup. This additional pressure renders unidirectional bonds bidirectional, promoting flow in the backward direction and local breakdown of diodicity.^39^

**Figure 2:**
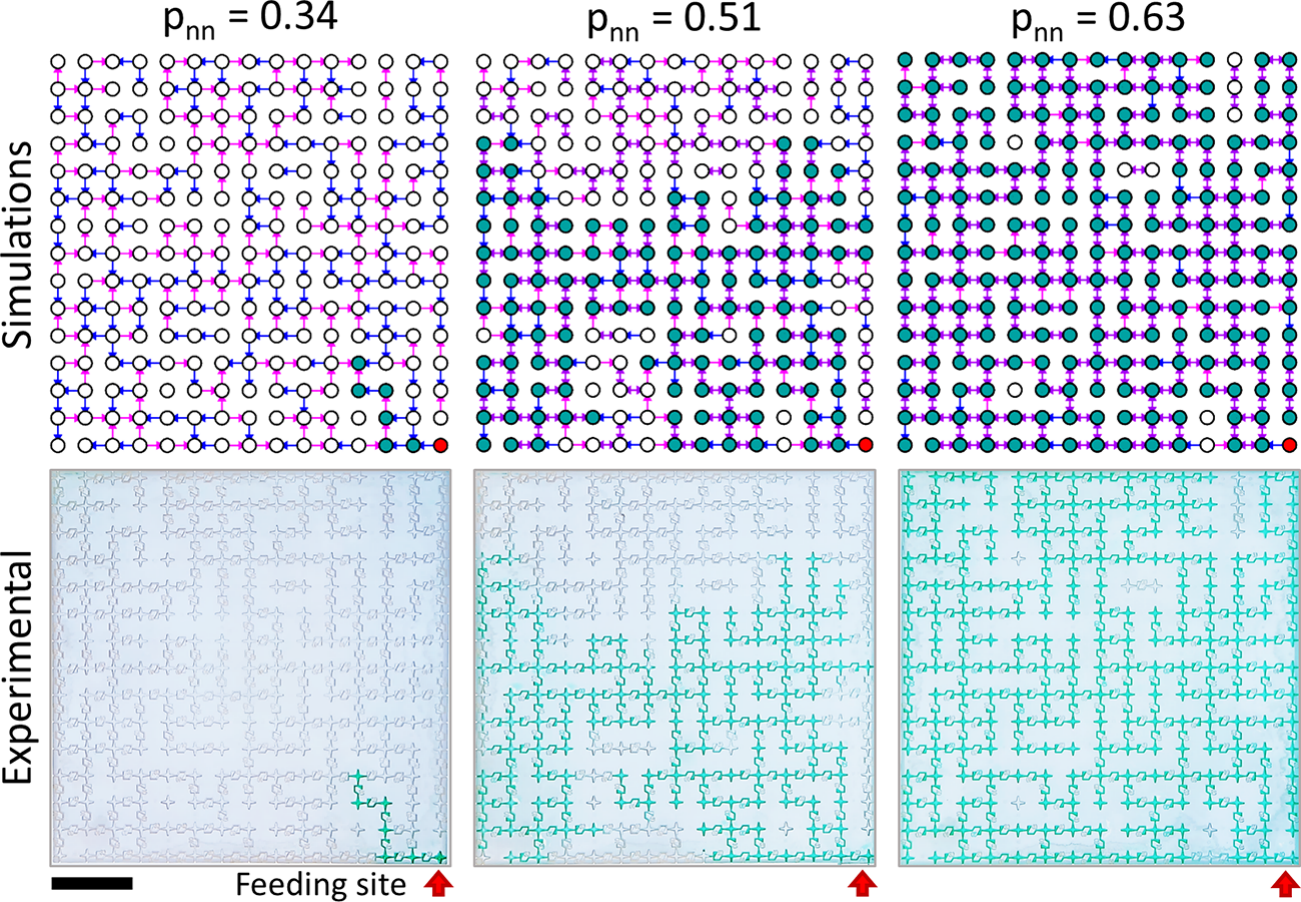
Simulations (top) and experiments (bottom) of final percolation states for increasing values of *p*_*nn*_, for a sample with *p*_0_ = 0.31, starting from the same feeding site which is highlighted in red. The scale bar corresponds to 3 cm. The percolation transition to a fully wetted network is clearly visible, and experiments show excellent agreement with the simulations.

We now examine the dependence of *f*, the fraction of occupied sites, on *p*_*nn*_, as shown in Figure 3. We observe the typical S-shape of percolation curves.^1^ The three curves, for the three different *p*_0_ values, show identical behavior and a notable degree of collapse. Simulations (circles) and experiments (squares) show excellent agreement. For each set of experiments, with a specific CA (i.e., *p*_*nn*_), the fraction of occupied sites is averaged by taking the median of all experiments. This was done to account for discrepancies in the outcomes of experiments with the same *p*_*nn*_ values but different feeding sites. In fact, not all sites on the border result necessarily in a similar connected path and, hence, a similar *f*. Using the median reflects the distribution of the results and gives each experiment the appropriate weight, according to how often a specific outcome occurred.

**Figure 3:**
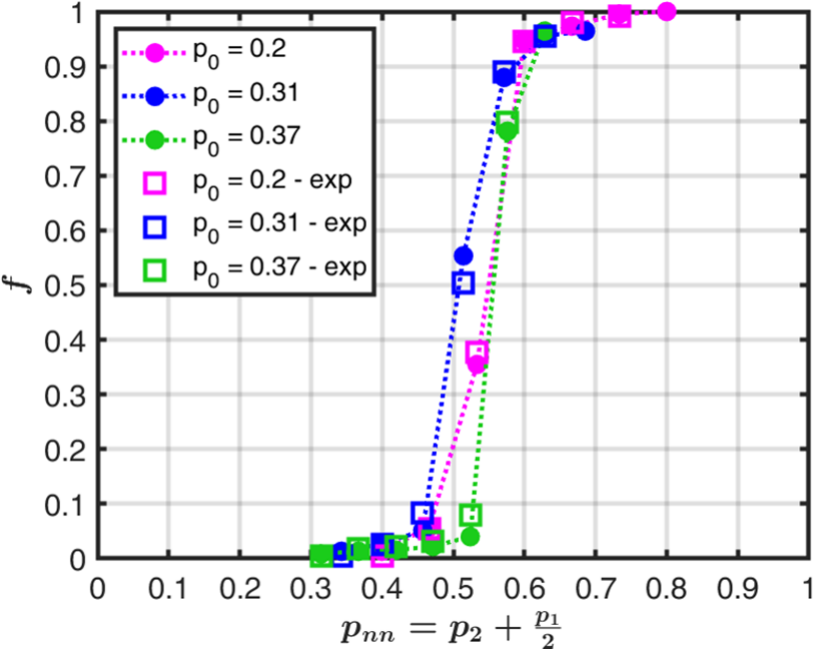
Fraction *f* of occupied sites as a function of *p*_*nn*_, for 15 × 15 networks with increasing values of *p*_0_ = 0.2, 0.31, and 0.37. Simulations (circles) and experiments (squares) show excellent agreement. Error bars in the experimental curves are smaller than the markers. The data collapse presents some scatter due to the limited system size.

The percolation threshold lies around , as expected for the square lattice.^29,40^ The variability seen in the data collapse is due to the moderate system size of the 3D-printed networks.^41,42^ The numerical simulations show that increasing the system size results in a better data collapse and a sharper percolation transition. This is manifested in Figure 4 by the magenta curves for system sizes of 15 × 15, 100 × 100 and 1000 × 1000. Each marker shape (diamond, circle and square) represents a different *p*_0_ value.

**Figure 4:**
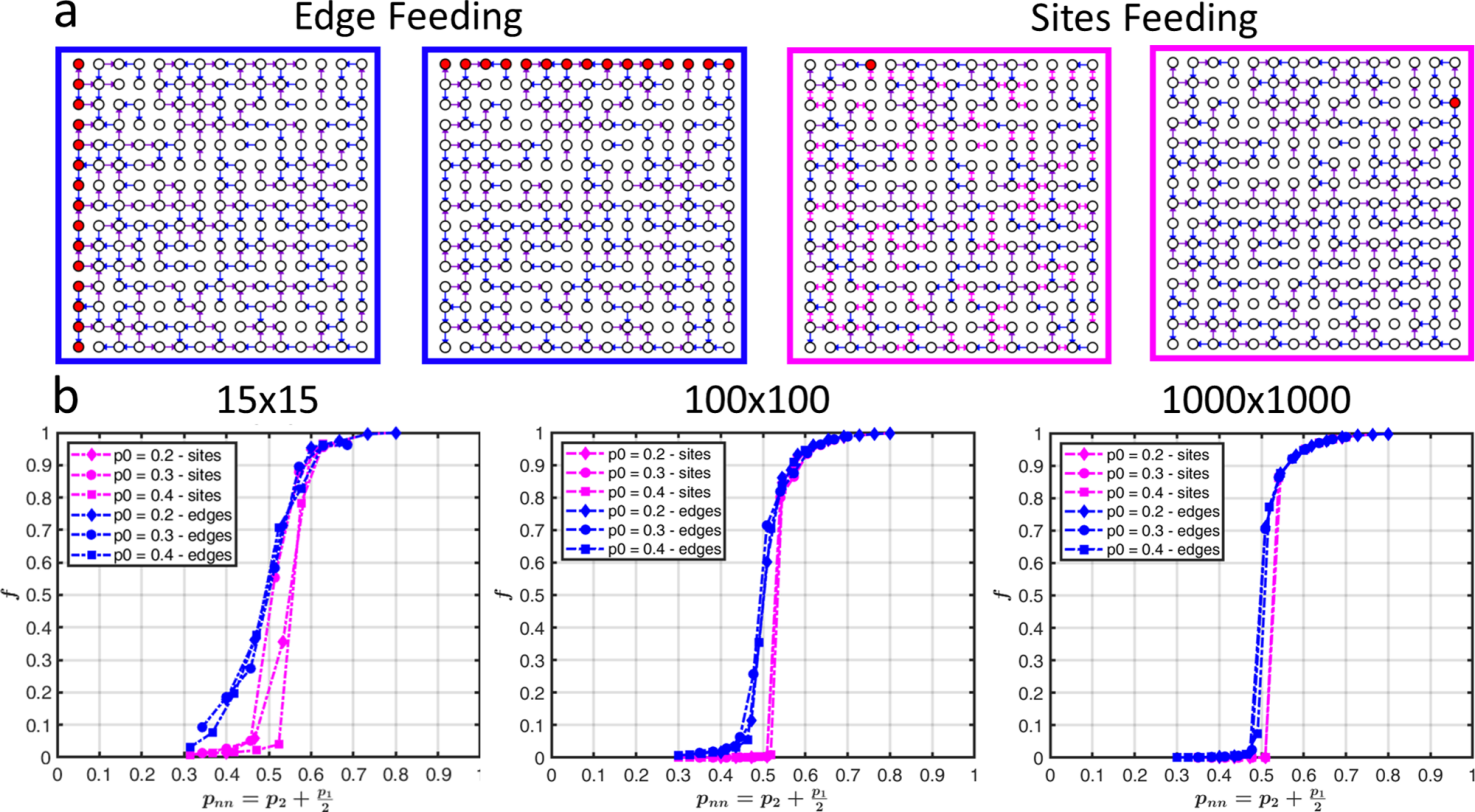
(a) Schematic representation of edge feeding (left) and site feeding (right). (b) Simulations showing the effect of system size and feeding method on the percolation curves, for 15 × 15, 100 × 100, and 1000 × 1000 isotropically directed networks. As the system size increases, the phase transition becomes sharper and the discrepancy between the two feeding methods becomes negligible.

Finally, we investigate the effect of different feeding protocols (Figure 4a) on the shape of the percolation curves for finite system sizes. The blue curves in Figure 4b represent percolation curves for increasing system size, obtained through numerical simulations, in which an entire edge of the network was fed rather than a single site on the edge. In this way, only four different initial conditions are averaged, in contrast to a number which increases with system perimeter in the case of a single feeding site. This feeding method results in a less sharp transition and a more symmetrical S-shape of the *f* versus *P_nn_* curve. The discrepancy between the two methods, more acute for small *p*_*nn*_ values, becomes less significant as the system size increases. Indeed, feeding the system through an entire edge results in an increased number of occupied sites for small *p*_*nn*_ values. This increase is significant for moderate (15 × 15) system sizes. but becomes minute for larger (1000 × 1000) networks.

In this work, we successfully conducted isotropically directed percolation experiments of flow in 2D networks of liquid diodes. Fine-tuning of the liquid diode geometry enabled us to control and manipulate the network’s statistical properties. Namely, to precisely control the amount of unidirectional and bidirectional bonds and, therefore, the flow patterns and propagation through the network. The excellent agreement between numerical simulations and experiments for finite-size systems of 15 × 15 sites validates the integrity of the simulations for larger scales. We gained new insights into the impact of the feeding method (initial conditions) on the percolation transition. When feeding the network from a single site, percolation curves are steeper, and the phase transition is more abrupt than feeding the system from an entire edge. This becomes less significant as we increase the network size and should be taken into account when analyzing or modeling real-life systems of moderate sizes. Additionally, we showed controlled passive transport of liquids in two dimensions over distances of about 15 cm. This is a testimony to the potential of liquid diodes not only as tools to investigate fundamental scientific questions but also to fabricate devices with a high degree of design flexibility and directionality. Further work, using different fabrication methods, may allow us to scale down the dimensions of the unit cells and sites, resulting in bigger networks featuring thousands of bonds.

## Materials and Methods

### 3D Printed Sample Design

The liquid diodes design is based on our previous work.^33^ The percentage of the pitch height was marked next to each unit cell for better visualization of the final sample. To design 2D networks, a computerized script was used to create a randomized isotropic distribution of diodes with various pitch heights for each value of *p*_0_ (number of vacancies). Namely, for every pitch height, the number of diodes in each orientation (right, left, up, and down) is identical. This way, from any random site within the network, there is a uniform probability of propagating in any direction.

The nodes of the network are designed as a 4-point star with filleted edges (Figure 1d,i). This way, each junction to the neighboring diode, when present, is a wedge, and the liquid is free to flow along the edges and around the corners. This proved to be more effective than a simple 90° cross design as the liquid would get potentially pinned at the intersections. The size of each sample is 15 × 15 sites. This was the most convenient size, in terms of fabrication and experiments. Each unit cell is a square with 4.88 mm long sides; the channels are 0.34 mm wide and 1.88 mm deep, the top border of the bulga is 1.46 mm, the lateral side of the bulga is 2.6 mm, and the top right angle of the bulga is 60°. These dimensions were chosen in order to remain within the capillary regime, as characterized by the capillary length. For water in air, this characteristic length corresponds to 2.7 mm. Down-scaling the structure poses difficulties in removing the supporting wax from within the channels after printing.

### Sample and Material Preparation

Samples were 3D-printed, using the ProJet MJP 2500 Series by 3D Systems (Rock Hill, South Carolina, USA), a multijet 3D printer. The material used for the printing is the VisiJet M2R-CL, a transparent polymer. Each sample, for each *p*_0_ value, was printed twice to check repeatability. After printing, the parts were cleaned to remove the supporting wax material. First, the support wax was dissolved in canola oil at 60 °C. The sample was then placed in a second heated oil bath for finer wax removal. The oil residues were later washed away in soapy water at 60 °C (all-purpose liquid detergent soap, soap to water volume ratio corresponds to 0.3:1). A small brush was used to gently clean the residues in narrow voids without damaging small features. Soap residues were thoroughly rinsed in deionized (DI) water, and the sample was dried in open air and then rinsed with ethanol and dried with an air gun. In all experiments, we used dyed DI water obtained by adding green food coloring (Maimon’s, Be’er Sheva, Israel) in a volume ratio of 0.05:1 (dye to water). Small quantities of all-purpose liquid detergent soap, ranging from a volume ratio of 0.02:1 to 0.07:1, were added to the water in order to decrease the native contact angle of the liquid. Six solutions with different contact angles were prepared and used in the experiments.

The cleanliness of the sample surface was crucial for good performance and minimal unexpected pinning of the liquid.^43−45^ After each experiment, samples were rinsed with DI water and ethanol to remove all soap residues and were then allowed to dry completely.

### Contact Angle Measurements

The CA of the liquids was measured using a contact angle goniometer, an OCA25 by DataPhysics Instruments GmbH (Filderstadt, Germany). For each of the six liquids, five measurements were taken of 2 μL drops from different areas of the surface. The CA was checked before each experiment.

### Percolation Experiments and Image Analysis

For each sample and for each CA (thus *p*_*nn*_ value), eight random sites along the outer edges, namely, two sites per edge, were picked as the feeding sites for percolation experiments, for a total of 144 experiments. Liquid of a known contact angle was slowly poured into the feeding site using a Transferpette S pipettes by BRAND GMBH + CO KG (Wertheim, Germany), in steps of 20 μL. This method enabled us to avoid pressure buildup around the feeding site, with consequent possible failures of neighboring diodes. Experiments were recorded from above using a Panasonic DC-S1 camera with Sigma 70 mm F2.8 DG Macro lens. Screenshots of videos and the fraction of occupied sites were obtained using a MATLAB script, adjusted from the one used in our previous work.^33^ The algorithm separates the final frame of the video into three RGB channels and subtracts the green from the red, highlighting only the liquid front with respect to the sample background. The frame is turned into a binary black-and-white image, which is divided into squares, each comprising a diode, a site, or a vacancy. For each square comprising a site, the mean of the central pixels is calculated. If the mean is larger than 0.3 (mostly white pixels), then the site is empty. If the mean is less than 0.3, the site is counted as filled by the liquid. For each set of experiments, for a specific *p*_*nn*_ value, the median of the fraction of occupied sites over the eight experiments was taken.

### Numerical Simulations

The numerical simulations reproduce the 3D-printed sample designs. For each CA, the distributions of the unidirectional and bidirectional bonds are well-defined, based on the phase diagram in Figure 1b. The *p*_*nn*_ value of each design is calculated and recorded. The network is then fed from a single site on the outer border, and the occupancy (fraction of occupied bonds) is computed using a pass/no-pass function according to the directionality of each bond. This is repeated for every site on the border, and the median of all initial conditions is calculated. The fraction *f* of occupied sites is then plotted against the corresponding *p*_*nn*_ value.

## Supporting Information Available

Figure S1. Schematics of the diode pitch (side-view), illustrating its function in creating a pressure barrier for preventing the liquid from propagating in the backward direction. Figure S2. Schematic representations of 15 × 15 networks with increasing *p*_0_ values, for increasing values of *p*_*nn*_ (PDF)
Video S1. Experimental: top-view of liquid (green) propagation in a 3D-printed network of liquid diodes with *p*_*nn*_ = 0.34 (speed ×15) (AVI)
Video S2. Experimental: top-view of liquid (green) propagation in a 3D-printed network of liquid diodes with *p*_*nn*_ = 0.51 (speed ×15) (AVI)
Video S3. Experimental: top-view of liquid (green) propagation in a 3D-printed network of liquid diodes with *p*_*nn*_ = 0.63 (speed ×15) (AVI)

## Supplementary Material

jz3c01885_si_004.pdf

jz3c01885_si_001.avi

jz3c01885_si_002.avi

jz3c01885_si_003.avi
