# Supplementary material for: Percolation in Networks of Liquid Diodes
Source: J Phys Chem Lett. 2023 Aug 22;14(34):7697–702. doi: 10.1021/acs.jpclett.3c01885 (PMC10476187; doi:10.1021/acs.jpclett.3c01885)
Supplement: Supplementary file 1 — jz3c01885_si_004.pdf [file jz3c01885_si_004.pdf]

Supporting Information

Percolation in Networks of Liquid Diodes

Camilla Sammartino,[†] Yair Shokef,[†,‡,¶,§] and Bat-El Pinchasik[*,†,‡]

†*School of Mechanical Engineering, Tel Aviv University, Tel Aviv 69978, Israel*

‡*Center for Physics and Chemistry of Living Systems, Tel Aviv University, Tel Aviv 69978, Israel*

¶*Center for Computational Molecular and Materials Science, and Center for Physics and Chemistry of Living Systems, Tel Aviv University, Tel Aviv 69978, Israel*

§*International Institute for Sustainability with Knotted Chiral Meta Matter, Hiroshima University, Japan*

E-mail: pinchasik@tauex.tau.ac.il

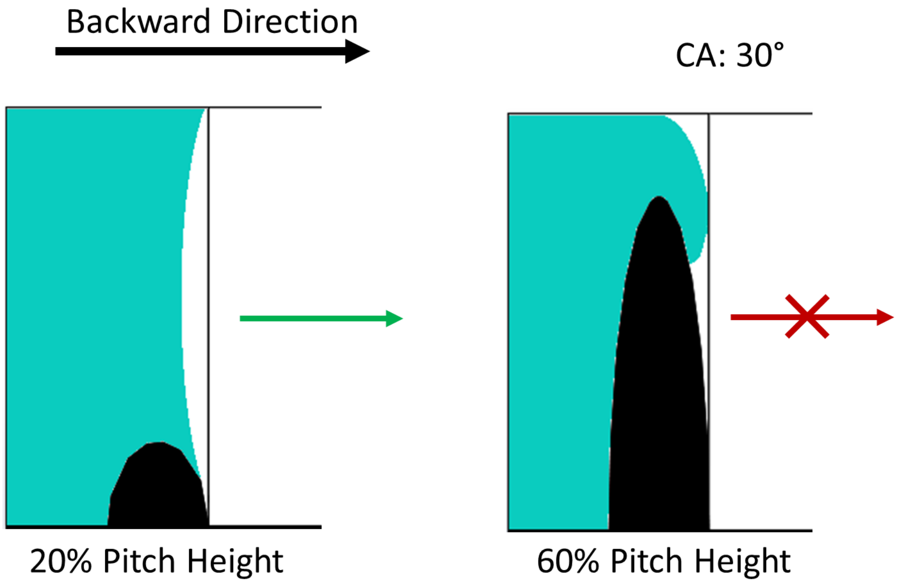

Figure 1: Schematics of the diode pitch (side-view), illustrating its function in creating a pressure barrier for preventing the liquid from propagating in the backward direction. A higher pitch results in a larger pressure barrier in the backward direction and improved diodicity.

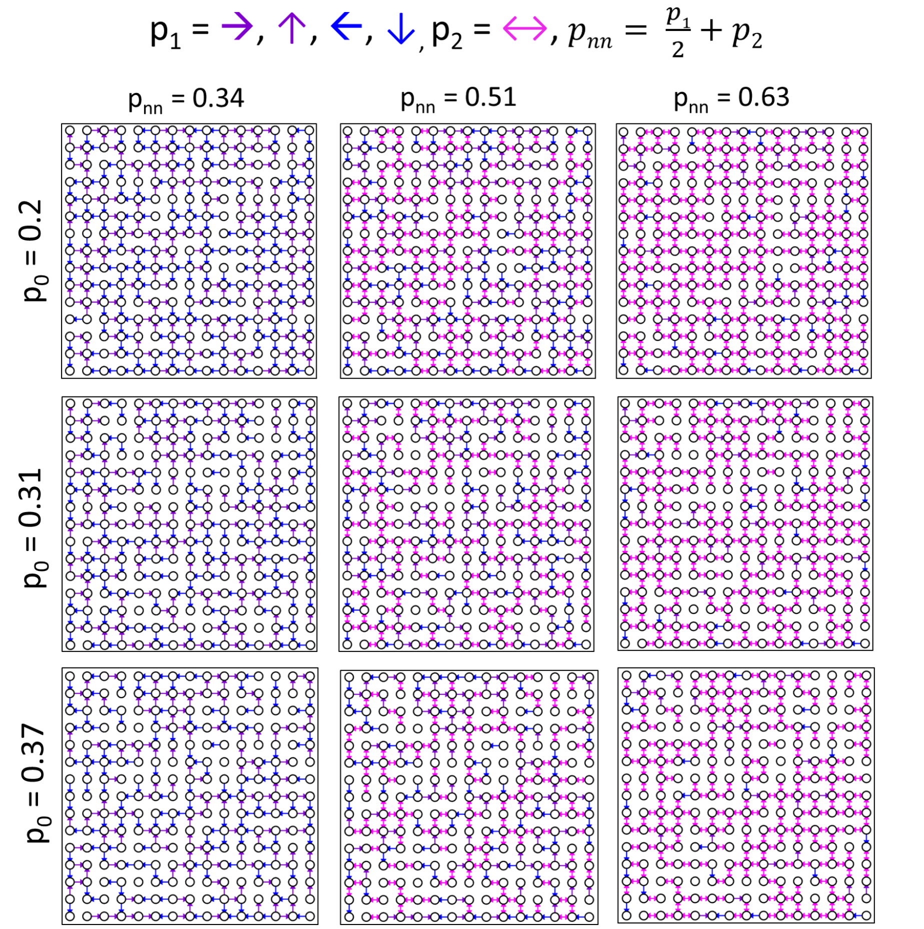

Figure 2: Schematic representations of 15x15 networks with increasing p_0 values, for increasing values of p_{nn}. As p_{nn} increases, a fraction of bonds becomes bidirectional (magenta double-pointed arrows), resulting in a bigger connected cluster.
